# (3R*,6R*,4′S*,8′R*,3′′R*,6′′R*)-3,3′′-Diisopropyl-6,6′′-dimethyl-2′,6′-di­phenyl­dispiro­[cyclo­hexane-1,4′-(3,7-dioxa-2,6-di­aza­bicyclo­[3.3.0]octa­ne)-8′,1′′-cyclo­hexa­ne]-2,2′′-dione

**DOI:** 10.1107/S1600536813017054

**Published:** 2013-06-26

**Authors:** Abdeslam Ansari, Lhou Majidi, Rachid Fihi, Jean-Claude Daran, Mohamed Azrour

**Affiliations:** a Laboratoire des Substances Naturelles et Synthèse et Dynamique Moléculaire, Faculté des Sciences et Techniques, Errachidia, Morocco; b Laboratoire de Chimie de Coordination, UPR-CNRS 8241, 205 route de Narbonne, 31077 Toulouse cedex, France; c Laboratoire de Chimie Physique des Matériaux, Faculté des Sciences et Techniques, BP 509, Errachidia, Morocco

## Abstract

The two oxazolidine rings (*A* and *B*) of the title compound, C_34_H_44_N_2_O_4_, display roughly half-chair conformations, which could be described as twisted on the C—O bond. Together, the fused oxazolidine rings have a butterfly shape, with the H atoms attached to the ring junction C atoms in a *cis* orientation. The cyclo­hexane rings of both *p*-menthone fragments display chair conformations. The absolute configuration could not be determined from the X-ray diffraction data, but the relative configuration of the stereocentres could be deduced.

## Related literature

For a related synthesis, see: Brüning *et al.* (1973 ▶); Tanka *et al.* (1972 ▶). For the properties of *p*-menthane derivatives, see: Ito *et al.* (2009 ▶); Kharchouf *et al.* (2011 ▶, 2012 ▶); Majidi *et al.* (2010 ▶); Clark (1990 ▶); Umemoko (1998 ▶); Boelens (1993 ▶); Wagner *et al.* (2004 ▶). For related structures, see: Iball *et al.* (1968 ▶, 1986 ▶); Aurich *et al.* (1989 ▶). For ring conformations, see: Cremer & Pople (1975 ▶).
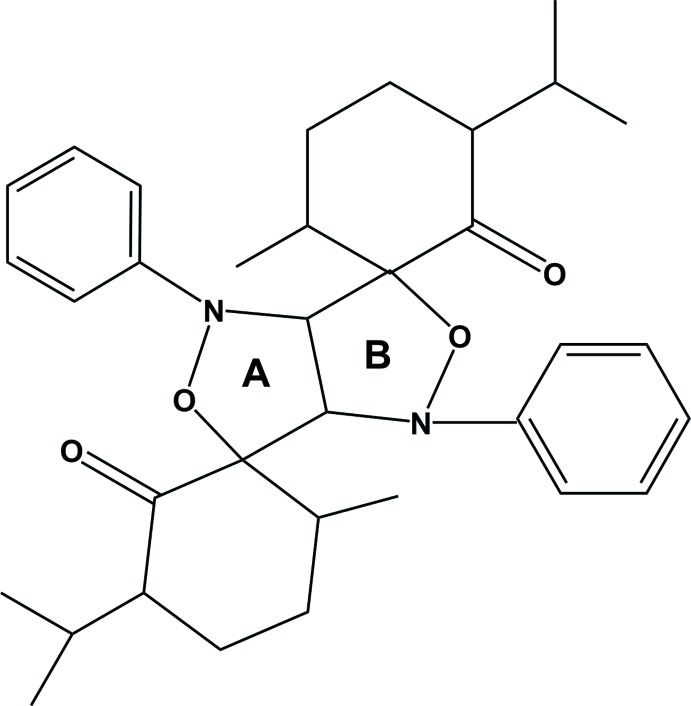


## Experimental

### 

#### Crystal data

C_34_H_44_N_2_O_4_
*M* *_r_* = 544.71
Orthorhombic, 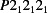

*a* = 9.5037 (6) Å
*b* = 12.4162 (10) Å
*c* = 24.8982 (18) Å
*V* = 2938.0 (4) Å^3^
*Z* = 4
Mo *K*α radiation
μ = 0.08 mm^−1^
*T* = 180 K
0.37 × 0.13 × 0.06 mm

#### Data collection

Agilent Xcalibur diffractometer
Absorption correction: multi-scan (SCALE3 ABSPACK in *CrysAlis PRO*; Agilent, 2010 ▶) *T*_min_ = 0.908, *T*_max_ = 1.000
24309 measured reflections
5963 independent reflections
3751 reflections with *I* > 2σ(*I*)
*R*_int_ = 0.098

#### Refinement

*R*[*F* ^2^ > 2σ(*F* ^2^)] = 0.045
*wR*(*F* ^2^) = 0.080
*S* = 0.87
5963 reflections
367 parameters
H-atom parameters constrained
Δρ_max_ = 0.17 e Å^−3^
Δρ_min_ = −0.19 e Å^−3^

### 

Data collection: *CrysAlis PRO* (Agilent, 2010 ▶); cell refinement: *CrysAlis PRO*; data reduction: *CrysAlis PRO*; program(s) used to solve structure: *SIR97* (Altomare *et al.*, 1999 ▶); program(s) used to refine structure: *SHELXL97* (Sheldrick, 2008 ▶); molecular graphics: *ORTEPIII* (Burnett & Johnson, 1996 ▶) and *ORTEP-3 for Windows* (Farrugia, 2012 ▶); software used to prepare material for publication: *SHELXL97*.

## Supplementary Material

Crystal structure: contains datablock(s) I, global. DOI: 10.1107/S1600536813017054/fy2097sup1.cif

Structure factors: contains datablock(s) I. DOI: 10.1107/S1600536813017054/fy2097Isup2.hkl

Additional supplementary materials:  crystallographic information; 3D view; checkCIF report

## Figures and Tables

**Fig. 1.**
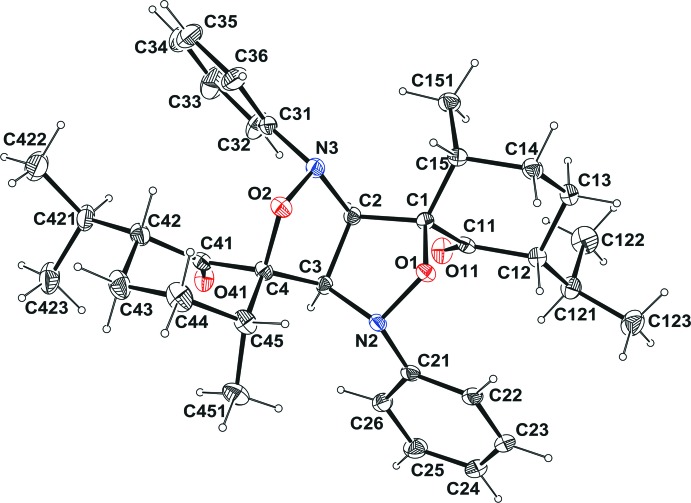
Molecular view of the title compound with the atom labeling scheme. Ellipsoids are drawn at the 30% probability level. H atoms are represented as small spheres of arbitrary radii.

**Fig. 2.**
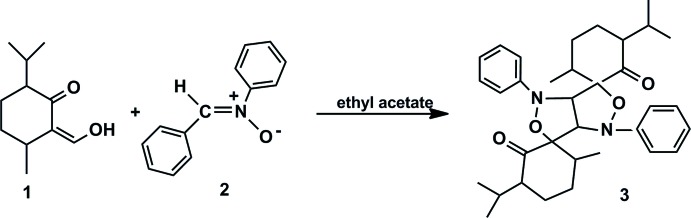
Scheme showing the synthetic pathway

## supplementary crystallographic information

### Comment

Synthesis of various *p*-menthane derivatives is studied extensively, with the goal to obtain biologically active and ecofriendly corrosion inhibitor compounds (Ito *et al.*, 2009; Kharchouf *et al.*, 2011; 2012; Majidi *et al.*, 2010). *p*-Menthan-3-one, 1 (Menthone) have become the key starting natural compound for the synthesis of a number of substances exhibiting various kinds of biological activity (Ito *et al.*, 2009). Menthone, a monoterpene ketone, occurs in nature and is widely present in high concentration in a few Mentha species, such as *Mentha specata aromentha* (Clark, 1990), *M*. *Avrvensis* (Umemoko, 1998) and the essential oils of pepperimint and other mint oils (Boelens, 1993). On the other hand, isoxazolidine rings are the frame of a number of natural products and antibiotics and are extensively used in the synthesis of a great many biologically important compounds (Wagner *et al.*, 2004). The goal of the present study was to obtain a new *p*-menthane derivative having two isoxazolidine moieties. This latter is of interest, because it can exhibit biological activity and has useful properties as precursor for synthesis.

The structure of the title compound is built up from two fused five membered oxazolidine rings sharing two C atoms to which *p*-menthone and phenyl rings are attached (Fig. 1). As observed in other diisooxazolidines (Iball *et al.*, 1968; 1986; Aurich *et al.*, 1989), the two oxazolidine rings display roughly half-chair conformation with the puckering parameters Q(2)= 0.366 (2) Å and φ= 350.2 (3)° for ring A (C1, C2, C3, O1, N2) and Q(2)= 0.360 (2) Å and φ(2= 347.7 (3)° for ring B (C2, C3, C4, O2, N3) (Cremer & Pople, 1975). They could be regarded as twisted on C1—O1 and C4—O2, respectively. This two fused rings have a butterfly shape with the H atoms attached to the C2—C3 edge in *cis* position. Both *p*-menthone fragments display a chair conformation with the puckering amplitudes of θ= 180.0 (2)°, φ= 74 (10)° and θ= 1.2 (2)°, φ= 314 (8)° respectively. The packing is stabilized only by van der Waals interactions.

### Experimental

2-Hydroxymethyle menthone (1) and n-diphenylnitrone (2) were prepared according to literature procedures (Brüning *et al.*, 1973; Tanka *et al.*, 1972).

To a mixture of 6 mmoles of 2-hydroxymethylene menthone and 6 mmoles of n-diphenylnitrone in 20 ml of ethyl acetate (Fig. 2) was added a catalytic amount of K_10 _(montmorillonite/FeIII). The reaction mixture was stirred for 48 h at 25°C and then filtered. The solvent was removed and the product was purified by recrystallization in ethanol (yield 67%). Single cristals were obtained by slow evaporation of an ethanol solution at room temperature.

### Refinement

In the absence of significant anomalous scattering, the absolute configuration could not be reliably determined, so any reference to the Flack parameter was removed.

All H atoms attached to C atoms were fixed geometrically and treated as riding with C—H = 1.0 Å (methine), 0.99 Å (methylene) or 0.98 Å (methyl), with *U*_iso_(H) = 1.2*U*_eq_(CH, CH_2_) or *U*_iso_(H) = 1.5*U*_eq_(CH_3_).

### Crystal data

**Table:**

C_34_H_44_N_2_O_4_	*F*(000) = 1176
*M**_r_* = 544.71	*D*_x_ = 1.231 Mg m^−^^3^
Orthorhombic, *P*2_1_2_1_2_1_	Mo *K*α radiation, λ = 0.71073 Å
Hall symbol: P 2ac 2ab	Cell parameters from 6434 reflections
*a* = 9.5037 (6) Å	θ = 3.0–28.6°
*b* = 12.4162 (10) Å	µ = 0.08 mm^−^^1^
*c* = 24.8982 (18) Å	*T* = 180 K
*V* = 2938.0 (4) Å^3^	Parallepiped, colourless
*Z* = 4	0.37 × 0.13 × 0.06 mm

### Data collection

**Table:**

Agilent Xcalibur diffractometer	5963 independent reflections
Radiation source: fine-focus sealed tube	3751 reflections with *I* > 2σ(*I*)
Graphite monochromator	*R*_int_ = 0.098
Detector resolution: 8.2632 pixels mm^-1^	θ_max_ = 26.4°, θ_min_ = 3.0°
ω scans	*h* = −11→10
Absorption correction: multi-scan (SCALE3 ABSPACK in *CrysAlis PRO*; Agilent, 2010)	*k* = −15→15
*T*_min_ = 0.908, *T*_max_ = 1.000	*l* = −31→31
24309 measured reflections	

### Refinement

**Table:**

Refinement on *F*^2^	Primary atom site location: structure-invariant direct methods
Least-squares matrix: full	Secondary atom site location: difference Fourier map
*R*[*F*^2^ > 2σ(*F*^2^)] = 0.045	Hydrogen site location: inferred from neighbouring sites
*wR*(*F*^2^) = 0.080	H-atom parameters constrained
*S* = 0.87	*w* = 1/[σ^2^(*F*_o_^2^) + (0.0302*P*)^2^] where *P* = (*F*_o_^2^ + 2*F*_c_^2^)/3
5963 reflections	(Δ/σ)_max_ = 0.001
367 parameters	Δρ_max_ = 0.17 e Å^−^^3^
0 restraints	Δρ_min_ = −0.19 e Å^−^^3^

### Special details

**Table:**

Geometry. All e.s.d.'s (except the e.s.d. in the dihedral angle between two l.s. planes) are estimated using the full covariance matrix. The cell e.s.d.'s are taken into account individually in the estimation of e.s.d.'s in distances, angles and torsion angles; correlations between e.s.d.'s in cell parameters are only used when they are defined by crystal symmetry. An approximate (isotropic) treatment of cell e.s.d.'s is used for estimating e.s.d.'s involving l.s. planes.
Refinement. Refinement of *F*^2^ against ALL reflections. The weighted *R*-factor *wR* and goodness of fit *S* are based on *F*^2^, conventional *R*-factors *R* are based on *F*, with *F* set to zero for negative *F*^2^. The threshold expression of *F*^2^ > σ(*F*^2^) is used only for calculating *R*-factors(gt) *etc*. and is not relevant to the choice of reflections for refinement. *R*-factors based on *F*^2^ are statistically about twice as large as those based on *F*, and *R*- factors based on ALL data will be even larger.

### Fractional atomic coordinates and isotropic or equivalent isotropic displacement parameters (Å^2^)

**Table:**

	*x*	*y*	*z*	*U*_iso_*/*U*_eq_	
C1	0.19364 (19)	0.00584 (18)	0.11958 (8)	0.0199 (5)	
C2	0.3120 (2)	0.07456 (17)	0.14271 (9)	0.0221 (5)	
H2	0.3371	0.0516	0.1800	0.026*	
C3	0.4346 (2)	0.05539 (17)	0.10324 (8)	0.0218 (5)	
H3	0.5146	0.0178	0.1214	0.026*	
C4	0.47632 (19)	0.16842 (17)	0.08623 (9)	0.0225 (5)	
C11	0.1960 (2)	−0.10828 (18)	0.14225 (9)	0.0238 (5)	
C12	0.09059 (19)	−0.18512 (17)	0.11807 (9)	0.0241 (5)	
H12	0.1106	−0.1858	0.0786	0.029*	
C13	−0.0568 (2)	−0.13708 (18)	0.12394 (9)	0.0300 (6)	
H13A	−0.1258	−0.1838	0.1054	0.036*	
H13B	−0.0826	−0.1342	0.1624	0.036*	
C14	−0.0626 (2)	−0.02464 (18)	0.10027 (10)	0.0306 (6)	
H14A	−0.1582	0.0053	0.1056	0.037*	
H14B	−0.0450	−0.0289	0.0611	0.037*	
C15	0.04500 (19)	0.05139 (17)	0.12565 (9)	0.0233 (5)	
H15	0.0404	0.1223	0.1067	0.028*	
C21	0.4178 (2)	−0.12024 (18)	0.05577 (8)	0.0240 (5)	
C22	0.3369 (2)	−0.18798 (19)	0.02411 (9)	0.0295 (6)	
H22	0.2577	−0.1598	0.0056	0.035*	
C23	0.3692 (2)	−0.2946 (2)	0.01916 (9)	0.0358 (6)	
H23	0.3107	−0.3401	−0.0019	0.043*	
C24	0.4860 (2)	−0.3371 (2)	0.04434 (10)	0.0397 (7)	
H24	0.5074	−0.4116	0.0416	0.048*	
C25	0.5706 (2)	−0.2691 (2)	0.07344 (10)	0.0385 (6)	
H25	0.6530	−0.2968	0.0900	0.046*	
C26	0.5387 (2)	−0.1620 (2)	0.07909 (9)	0.0320 (6)	
H26	0.5994	−0.1163	0.0990	0.038*	
C31	0.3156 (2)	0.25848 (19)	0.18430 (10)	0.0290 (6)	
C32	0.3771 (3)	0.2242 (2)	0.23146 (10)	0.0455 (7)	
H32	0.4013	0.1505	0.2361	0.055*	
C33	0.4036 (3)	0.2983 (3)	0.27216 (12)	0.0649 (9)	
H33	0.4469	0.2749	0.3045	0.078*	
C34	0.3683 (3)	0.4037 (3)	0.26628 (13)	0.0607 (9)	
H34	0.3854	0.4533	0.2946	0.073*	
C35	0.3081 (3)	0.4380 (2)	0.21937 (13)	0.0576 (8)	
H35	0.2859	0.5121	0.2147	0.069*	
C36	0.2796 (2)	0.3658 (2)	0.17905 (11)	0.0477 (7)	
H36	0.2346	0.3898	0.1472	0.057*	
C41	0.5902 (2)	0.21326 (18)	0.12434 (9)	0.0244 (5)	
C42	0.6212 (2)	0.33086 (18)	0.11805 (9)	0.0318 (6)	
H42	0.5296	0.3696	0.1222	0.038*	
C43	0.6684 (2)	0.3473 (2)	0.05962 (10)	0.0437 (7)	
H43A	0.6837	0.4251	0.0530	0.052*	
H43B	0.7589	0.3097	0.0538	0.052*	
C44	0.5597 (2)	0.3048 (2)	0.01977 (10)	0.0423 (7)	
H44A	0.4722	0.3474	0.0233	0.051*	
H44B	0.5958	0.3148	−0.0172	0.051*	
C45	0.5259 (2)	0.18599 (19)	0.02852 (8)	0.0316 (6)	
H45	0.4465	0.1665	0.0040	0.038*	
C121	0.1084 (2)	−0.30106 (18)	0.13674 (9)	0.0323 (6)	
H121	0.2116	−0.3163	0.1382	0.039*	
C122	0.0492 (3)	−0.3200 (2)	0.19291 (10)	0.0504 (7)	
H12A	−0.0523	−0.3058	0.1928	0.076*	
H12B	0.0955	−0.2714	0.2184	0.076*	
H12C	0.0662	−0.3948	0.2036	0.076*	
C123	0.0439 (2)	−0.37914 (19)	0.09676 (11)	0.0463 (7)	
H12D	−0.0577	−0.3665	0.0945	0.069*	
H12E	0.0612	−0.4532	0.1086	0.069*	
H12F	0.0865	−0.3681	0.0614	0.069*	
C151	0.0135 (2)	0.06976 (19)	0.18542 (9)	0.0314 (6)	
H15A	0.0318	0.0033	0.2054	0.047*	
H15B	−0.0855	0.0903	0.1898	0.047*	
H15C	0.0739	0.1275	0.1992	0.047*	
C421	0.7198 (2)	0.37487 (19)	0.16169 (11)	0.0410 (7)	
H421	0.6917	0.3406	0.1964	0.049*	
C422	0.7019 (3)	0.4951 (2)	0.16820 (14)	0.0645 (9)	
H42A	0.7648	0.5211	0.1966	0.097*	
H42B	0.6042	0.5111	0.1779	0.097*	
H42C	0.7252	0.5312	0.1343	0.097*	
C423	0.8732 (2)	0.3466 (2)	0.15191 (12)	0.0554 (8)	
H42D	0.9069	0.3843	0.1198	0.083*	
H42E	0.8822	0.2687	0.1466	0.083*	
H42F	0.9296	0.3685	0.1830	0.083*	
C451	0.6509 (2)	0.1136 (2)	0.01454 (9)	0.0402 (7)	
H45A	0.7289	0.1286	0.0392	0.060*	
H45B	0.6809	0.1278	−0.0224	0.060*	
H45C	0.6230	0.0379	0.0180	0.060*	
N2	0.37834 (15)	−0.00963 (14)	0.05922 (7)	0.0228 (4)	
N3	0.27983 (16)	0.18972 (14)	0.14028 (7)	0.0263 (4)	
O1	0.22708 (13)	0.00147 (12)	0.06227 (5)	0.0229 (3)	
O2	0.34705 (13)	0.22877 (12)	0.09175 (6)	0.0267 (4)	
O11	0.27485 (15)	−0.13285 (13)	0.17847 (6)	0.0369 (4)	
O41	0.65421 (14)	0.15395 (13)	0.15382 (6)	0.0333 (4)	

### Atomic displacement parameters (Å^2^)

**Table:**

	*U*^11^	*U*^22^	*U*^33^	*U*^12^	*U*^13^	*U*^23^
C1	0.0225 (11)	0.0246 (12)	0.0126 (11)	−0.0020 (9)	−0.0001 (9)	0.0004 (11)
C2	0.0219 (11)	0.0222 (13)	0.0221 (13)	−0.0006 (9)	−0.0012 (9)	−0.0006 (11)
C3	0.0216 (11)	0.0240 (13)	0.0199 (12)	−0.0030 (9)	−0.0015 (10)	−0.0009 (11)
C4	0.0190 (10)	0.0247 (13)	0.0239 (12)	0.0007 (9)	−0.0005 (9)	0.0036 (10)
C11	0.0192 (11)	0.0326 (14)	0.0198 (12)	0.0032 (10)	0.0036 (10)	0.0019 (11)
C12	0.0254 (11)	0.0250 (13)	0.0219 (13)	−0.0010 (10)	−0.0023 (9)	0.0036 (11)
C13	0.0251 (12)	0.0310 (15)	0.0340 (14)	−0.0036 (10)	−0.0006 (10)	−0.0012 (12)
C14	0.0241 (12)	0.0359 (16)	0.0320 (14)	0.0019 (11)	−0.0035 (10)	−0.0015 (12)
C15	0.0248 (12)	0.0252 (13)	0.0200 (12)	0.0021 (10)	0.0004 (10)	0.0009 (11)
C21	0.0238 (11)	0.0293 (14)	0.0189 (12)	0.0003 (10)	0.0059 (10)	−0.0024 (12)
C22	0.0270 (12)	0.0358 (15)	0.0256 (14)	0.0010 (11)	0.0032 (10)	−0.0089 (12)
C23	0.0333 (13)	0.0376 (17)	0.0366 (15)	−0.0031 (12)	0.0067 (11)	−0.0171 (13)
C24	0.0388 (14)	0.0302 (15)	0.0500 (17)	0.0016 (12)	0.0097 (12)	−0.0135 (14)
C25	0.0353 (14)	0.0361 (16)	0.0440 (17)	0.0091 (12)	0.0002 (12)	−0.0051 (14)
C26	0.0286 (12)	0.0328 (15)	0.0347 (15)	0.0021 (11)	−0.0005 (11)	−0.0090 (12)
C31	0.0205 (12)	0.0317 (15)	0.0348 (15)	−0.0031 (10)	0.0007 (10)	−0.0104 (13)
C32	0.0694 (18)	0.0360 (17)	0.0312 (15)	−0.0123 (14)	−0.0038 (14)	−0.0065 (14)
C33	0.094 (2)	0.063 (2)	0.0376 (18)	−0.0190 (18)	−0.0093 (16)	−0.0116 (18)
C34	0.068 (2)	0.056 (2)	0.059 (2)	−0.0174 (17)	0.0087 (17)	−0.0305 (19)
C35	0.0512 (17)	0.0399 (18)	0.082 (2)	0.0032 (14)	−0.0046 (17)	−0.0272 (18)
C36	0.0443 (15)	0.0363 (17)	0.063 (2)	0.0086 (13)	−0.0112 (13)	−0.0184 (16)
C41	0.0216 (12)	0.0271 (14)	0.0244 (13)	0.0013 (10)	0.0041 (10)	0.0039 (12)
C42	0.0248 (12)	0.0269 (14)	0.0437 (16)	0.0005 (10)	0.0001 (11)	0.0061 (12)
C43	0.0361 (13)	0.0358 (15)	0.0592 (18)	−0.0059 (12)	0.0021 (13)	0.0159 (15)
C44	0.0451 (14)	0.0447 (18)	0.0372 (16)	−0.0022 (13)	0.0030 (12)	0.0203 (14)
C45	0.0308 (12)	0.0414 (16)	0.0225 (13)	−0.0021 (11)	−0.0001 (10)	0.0096 (12)
C121	0.0379 (13)	0.0263 (14)	0.0327 (14)	−0.0034 (10)	0.0001 (11)	0.0063 (12)
C122	0.0646 (17)	0.0433 (17)	0.0431 (17)	−0.0046 (14)	0.0067 (13)	0.0184 (14)
C123	0.0570 (16)	0.0292 (16)	0.0526 (18)	−0.0078 (13)	0.0031 (13)	−0.0004 (15)
C151	0.0320 (13)	0.0353 (15)	0.0269 (14)	0.0010 (11)	0.0049 (11)	−0.0020 (12)
C421	0.0323 (14)	0.0270 (14)	0.0637 (18)	−0.0071 (11)	−0.0068 (12)	−0.0021 (14)
C422	0.0462 (17)	0.0366 (17)	0.111 (3)	−0.0039 (14)	−0.0063 (17)	−0.015 (2)
C423	0.0355 (15)	0.0376 (16)	0.093 (2)	−0.0077 (13)	−0.0169 (14)	0.0026 (17)
C451	0.0358 (13)	0.0536 (18)	0.0313 (15)	0.0012 (12)	0.0106 (11)	0.0028 (14)
N2	0.0167 (9)	0.0292 (11)	0.0227 (10)	−0.0012 (8)	0.0007 (8)	−0.0035 (10)
N3	0.0275 (10)	0.0274 (11)	0.0240 (11)	−0.0036 (8)	0.0034 (8)	−0.0001 (10)
O1	0.0197 (8)	0.0323 (9)	0.0166 (8)	0.0005 (7)	−0.0002 (6)	0.0007 (8)
O2	0.0239 (8)	0.0286 (9)	0.0276 (9)	0.0028 (7)	0.0002 (7)	0.0057 (8)
O11	0.0371 (9)	0.0362 (10)	0.0372 (10)	−0.0018 (8)	−0.0120 (8)	0.0095 (9)
O41	0.0339 (9)	0.0284 (9)	0.0375 (10)	−0.0036 (7)	−0.0097 (7)	0.0073 (8)

### Geometric parameters (Å, º)

**Table:**

C1—O1	1.463 (2)	C34—C35	1.369 (4)
C1—C2	1.525 (3)	C34—H34	0.9500
C1—C11	1.525 (3)	C35—C36	1.373 (3)
C1—C15	1.529 (3)	C35—H35	0.9500
C2—N3	1.463 (3)	C36—H36	0.9500
C2—C3	1.543 (3)	C41—O41	1.204 (2)
C2—H2	1.0000	C41—C42	1.498 (3)
C3—N2	1.463 (3)	C42—C421	1.536 (3)
C3—C4	1.519 (3)	C42—C43	1.536 (3)
C3—H3	1.0000	C42—H42	1.0000
C4—O2	1.446 (2)	C43—C44	1.526 (3)
C4—C45	1.528 (3)	C43—H43A	0.9900
C4—C41	1.543 (3)	C43—H43B	0.9900
C11—O11	1.212 (2)	C44—C45	1.525 (3)
C11—C12	1.509 (3)	C44—H44A	0.9900
C12—C121	1.522 (3)	C44—H44B	0.9900
C12—C13	1.530 (3)	C45—C451	1.530 (3)
C12—H12	1.0000	C45—H45	1.0000
C13—C14	1.516 (3)	C121—C123	1.519 (3)
C13—H13A	0.9900	C121—C122	1.526 (3)
C13—H13B	0.9900	C121—H121	1.0000
C14—C15	1.529 (3)	C122—H12A	0.9800
C14—H14A	0.9900	C122—H12B	0.9800
C14—H14B	0.9900	C122—H12C	0.9800
C15—C151	1.535 (3)	C123—H12D	0.9800
C15—H15	1.0000	C123—H12E	0.9800
C21—C22	1.386 (3)	C123—H12F	0.9800
C21—C26	1.388 (3)	C151—H15A	0.9800
C21—N2	1.426 (3)	C151—H15B	0.9800
C22—C23	1.365 (3)	C151—H15C	0.9800
C22—H22	0.9500	C421—C422	1.512 (3)
C23—C24	1.380 (3)	C421—C423	1.519 (3)
C23—H23	0.9500	C421—H421	1.0000
C24—C25	1.372 (3)	C422—H42A	0.9800
C24—H24	0.9500	C422—H42B	0.9800
C25—C26	1.370 (3)	C422—H42C	0.9800
C25—H25	0.9500	C423—H42D	0.9800
C26—H26	0.9500	C423—H42E	0.9800
C31—C32	1.379 (3)	C423—H42F	0.9800
C31—C36	1.382 (3)	C451—H45A	0.9800
C31—N3	1.430 (3)	C451—H45B	0.9800
C32—C33	1.392 (4)	C451—H45C	0.9800
C32—H32	0.9500	N2—O1	1.4461 (19)
C33—C34	1.358 (4)	N3—O2	1.450 (2)
C33—H33	0.9500		
			
O1—C1—C2	103.24 (15)	C35—C36—H36	119.6
O1—C1—C11	108.86 (17)	C31—C36—H36	119.6
C2—C1—C11	111.67 (16)	O41—C41—C42	124.1 (2)
O1—C1—C15	108.11 (15)	O41—C41—C4	120.54 (19)
C2—C1—C15	115.93 (18)	C42—C41—C4	115.16 (19)
C11—C1—C15	108.68 (16)	C41—C42—C421	113.1 (2)
N3—C2—C1	112.15 (17)	C41—C42—C43	106.6 (2)
N3—C2—C3	106.39 (16)	C421—C42—C43	116.40 (18)
C1—C2—C3	103.31 (16)	C41—C42—H42	106.7
N3—C2—H2	111.5	C421—C42—H42	106.7
C1—C2—H2	111.5	C43—C42—H42	106.7
C3—C2—H2	111.5	C44—C43—C42	111.85 (18)
N2—C3—C4	113.36 (17)	C44—C43—H43A	109.2
N2—C3—C2	106.65 (15)	C42—C43—H43A	109.2
C4—C3—C2	103.44 (16)	C44—C43—H43B	109.2
N2—C3—H3	111.0	C42—C43—H43B	109.2
C4—C3—H3	111.0	H43A—C43—H43B	107.9
C2—C3—H3	111.0	C45—C44—C43	112.6 (2)
O2—C4—C3	103.33 (15)	C45—C44—H44A	109.1
O2—C4—C45	106.12 (16)	C43—C44—H44A	109.1
C3—C4—C45	118.35 (18)	C45—C44—H44B	109.1
O2—C4—C41	110.52 (16)	C43—C44—H44B	109.1
C3—C4—C41	110.18 (17)	H44A—C44—H44B	107.8
C45—C4—C41	108.08 (16)	C44—C45—C4	109.72 (19)
O11—C11—C12	123.3 (2)	C44—C45—C451	111.84 (18)
O11—C11—C1	121.21 (19)	C4—C45—C451	111.68 (18)
C12—C11—C1	115.48 (18)	C44—C45—H45	107.8
C11—C12—C121	113.74 (17)	C4—C45—H45	107.8
C11—C12—C13	108.86 (18)	C451—C45—H45	107.8
C121—C12—C13	116.18 (17)	C123—C121—C12	111.02 (18)
C11—C12—H12	105.7	C123—C121—C122	110.7 (2)
C121—C12—H12	105.7	C12—C121—C122	112.6 (2)
C13—C12—H12	105.7	C123—C121—H121	107.4
C14—C13—C12	110.80 (17)	C12—C121—H121	107.4
C14—C13—H13A	109.5	C122—C121—H121	107.4
C12—C13—H13A	109.5	C121—C122—H12A	109.5
C14—C13—H13B	109.5	C121—C122—H12B	109.5
C12—C13—H13B	109.5	H12A—C122—H12B	109.5
H13A—C13—H13B	108.1	C121—C122—H12C	109.5
C13—C14—C15	112.55 (18)	H12A—C122—H12C	109.5
C13—C14—H14A	109.1	H12B—C122—H12C	109.5
C15—C14—H14A	109.1	C121—C123—H12D	109.5
C13—C14—H14B	109.1	C121—C123—H12E	109.5
C15—C14—H14B	109.1	H12D—C123—H12E	109.5
H14A—C14—H14B	107.8	C121—C123—H12F	109.5
C14—C15—C1	110.42 (17)	H12D—C123—H12F	109.5
C14—C15—C151	111.22 (17)	H12E—C123—H12F	109.5
C1—C15—C151	109.34 (17)	C15—C151—H15A	109.5
C14—C15—H15	108.6	C15—C151—H15B	109.5
C1—C15—H15	108.6	H15A—C151—H15B	109.5
C151—C15—H15	108.6	C15—C151—H15C	109.5
C22—C21—C26	118.0 (2)	H15A—C151—H15C	109.5
C22—C21—N2	118.23 (19)	H15B—C151—H15C	109.5
C26—C21—N2	123.5 (2)	C422—C421—C423	110.71 (19)
C23—C22—C21	121.0 (2)	C422—C421—C42	111.0 (2)
C23—C22—H22	119.5	C423—C421—C42	113.0 (2)
C21—C22—H22	119.5	C422—C421—H421	107.3
C22—C23—C24	120.7 (2)	C423—C421—H421	107.3
C22—C23—H23	119.6	C42—C421—H421	107.3
C24—C23—H23	119.6	C421—C422—H42A	109.5
C25—C24—C23	118.4 (2)	C421—C422—H42B	109.5
C25—C24—H24	120.8	H42A—C422—H42B	109.5
C23—C24—H24	120.8	C421—C422—H42C	109.5
C26—C25—C24	121.4 (2)	H42A—C422—H42C	109.5
C26—C25—H25	119.3	H42B—C422—H42C	109.5
C24—C25—H25	119.3	C421—C423—H42D	109.5
C25—C26—C21	120.2 (2)	C421—C423—H42E	109.5
C25—C26—H26	119.9	H42D—C423—H42E	109.5
C21—C26—H26	119.9	C421—C423—H42F	109.5
C32—C31—C36	118.9 (2)	H42D—C423—H42F	109.5
C32—C31—N3	124.7 (2)	H42E—C423—H42F	109.5
C36—C31—N3	116.4 (2)	C45—C451—H45A	109.5
C31—C32—C33	119.5 (3)	C45—C451—H45B	109.5
C31—C32—H32	120.3	H45A—C451—H45B	109.5
C33—C32—H32	120.3	C45—C451—H45C	109.5
C34—C33—C32	120.9 (3)	H45A—C451—H45C	109.5
C34—C33—H33	119.5	H45B—C451—H45C	109.5
C32—C33—H33	119.5	C21—N2—O1	110.86 (14)
C33—C34—C35	119.7 (3)	C21—N2—C3	118.73 (17)
C33—C34—H34	120.2	O1—N2—C3	105.74 (14)
C35—C34—H34	120.2	C31—N3—O2	109.51 (15)
C34—C35—C36	120.2 (3)	C31—N3—C2	120.11 (18)
C34—C35—H35	119.9	O2—N3—C2	105.61 (15)
C36—C35—H35	119.9	N2—O1—C1	105.71 (13)
C35—C36—C31	120.8 (3)	C4—O2—N3	106.28 (14)
